# Redo off-pump coronary artery bypass grafting via left thoracotomy

**DOI:** 10.1186/1749-8090-8-S1-P126

**Published:** 2013-09-11

**Authors:** I Duvan, M Kurtoglu, S Ates, BE Onuk, P Sungur, IS Karacan

**Affiliations:** 1 Department of Cardiovascular Surgery, Güven Hospital, Ankara, Turkey

## Background

In this study we reviewed our experience in a meticulously selected group of patients undergoing redo off-pump CABG from descending aorta to circumflex artery and branches retrospectively.

## Methods

Between January 2001 and May 2013 thirty patients underwent redo off-pump CABG from descending aorta to circumflex artery and branches via left thoracotomy at our hospital. Of the thirty patients, 26 were male (86.6%) and 4 were female (13.4%) with a mean age of 60.5 years. All patients had patent LIMA-LAD anastomosis. Left thoracotomy was performed through the fifth intercostal space. Saphenous vein (6) or radial artery (24) was prepared as grafts at the same time with thoracotomy from the contralateral extremity without any positional problem.

## Results

Main reasons for operation in this group of patients was new lesion formation in 24 (80%) and graft occlusion in 12 (23.3%), both in 6 (20%). Average operation time was 140.17 minutes, respiratory assist time was 4.9 hours, ICU period was 21.53 hours, hospital stay was 4.6 days. Thirty-six bypasses were performed. Follow up period was 47.8 months. Six patients were lost in follow-up whereas four patients were dead. Twenty were alive and well without any cardiac problems.

## Conclusion

Redo off-pump CABG via thoracotomy provides a safe and effective surgical approach to the patients who require revascularization of circumflex artery and branches. Therefore if the target area of revascularization is circumflex artery, left thoracotomy avoids such risks of resternotomy. Avoiding risks of resternotomy and CPB could possibly reduce the morbidity and hospital cost.

